# Step Length Estimation Using the RSSI Method in Walking and Jogging Scenarios

**DOI:** 10.3390/s22041640

**Published:** 2022-02-19

**Authors:** Zanru Yang, Le Chung Tran, Farzad Safaei

**Affiliations:** School of Electrical, Computer and Telecommunications Engineering, University of Wollongong, Wollongong, NSW 2522, Australia; lctran@uow.edu.au (L.C.T.); farzad@uow.edu.au (F.S.)

**Keywords:** data histogram, distance estimation, gait speed, on-ankle path loss, RSSI, step length estimation, strike length estimation, two-term Gaussian distribution

## Abstract

In this paper, human step length was estimated based on wireless channel properties and the received signal strength indicator (RSSI) method. Path loss between two ankles of the person under test was converted from the RSSI, which was measured using our developed wearable transceivers with embedded micro-controllers in four scenarios, namely indoor walking, outdoor walking, indoor jogging, and outdoor jogging. For brevity, we call it on-ankle path loss. The histogram of the on-ankle path loss showed clearly that there were two humps, where the second hump was closely related to the maximum path loss, which, in turn, corresponded to the step length. This histogram can be well approximated by a two-term Gaussian fitting curve model. Based on the histogram of the experimental data and the two-term Gaussian fitting curve, we propose a novel filtering technique to filter out the path loss outliers, which helps set up the upper and lower thresholds of the path loss values used for the step length estimation. In particular, the upper threshold was found to be on the right side of the second Gaussian hump, and its value was a function of the mean value and the standard deviation of the second Gaussian hump. Meanwhile, the lower threshold lied on the left side of the second hump and was determined at the point where the survival rate of the measured data fell to 0.68, i.e., the cumulative distribution function (CDF) approached 0.32. The experimental data showed that the proposed filtering technique resulted in high accuracy in step length estimation with errors of only 10.15 mm for the indoor walking, 4.40 mm for the indoor jogging, 4.81 mm for the outdoor walking, and 10.84 mm for the outdoor jogging scenarios, respectively.

## 1. Introduction

Step length (or stride length) plays an important role in addressing the issue of human health conditions, especially for seniors. It is an indicator that predicts accidental falls and fall-related injury in the elderly [1], which may cause fatality [2]. A reduced step length has been found to be associated with the increased dependence, mortality, and institutionalization of older people [3]. The variability of the step length also indicates the integrity of gray matter, which is closely related to personal memory and executive functions [4]. Furthermore, step length is one of the significant components in gait patterns. It can be converted to gait speed, which is useful in predicting life expectancy [5]. Therefore, monitoring the human step length is a vital topic that is worthy of investigating.

The estimation of the step length can be traced back to the problem of distance estimation. Although distance estimation has been intensively researched for general communication systems, there are few papers explicitly researching the human step length in daily activities, such as walking and jogging, in both indoor and outdoor environments. Moreover, as mentioned in more detail in the next section of this paper, the existing publications that address the estimation of step length either have modest accuracy or follow privacy-invasive, health-concerning, and strictly space-confined approaches. Specifically, camera-based technologies [6,7] are privacy-invasive and prone to error as they may record images or video footage of the participants. The camera-based methods also require a specific experimental setting because any obstacle appearing between the camera and the person under test can cause measurement errors. Meanwhile, laser-based methods [8] may arouse health concerns because a long-time exposure to lasers in these methods may cause some health hazards. On the other hand, sensing mats [9,10,11] have been well adopted to improve the safety of patients, especially the disabled and those with disorders. However, the sensing mat approach is confined to particular spaces, such as clinics, hospitals, or a specific laboratory setting where the sensing mat is laid, because the person under test must walk or run on this mat. Therefore, a more-accurate, less-invasive, less-health-concerning, and less-space-confined, but also cost-efficient technique for step length estimations in daily activities is still missing.

Thus, this paper aimed to estimate the step length based on the received signal strength indicator (RSSI) method in both walking and jogging activities in indoor and outdoor scenarios. The RSSI has been widely employed in distance estimation, and it might provide reliable performance [12,13,14,15,16,17], especially for measurements in line-of-sight (LOS) paths over short distances, such as the step length measurements in this paper. The step length in this paper refers to the average distance between two ankles of the person under test when the person is walking or jogging at a normal and equal pace. Unlike our previous work in [18], which only considered a static environment, this paper undertook experiments in actual moving activities. In particular, in this paper, we propose a novel filtering technique to be applied along with the empirical path loss model proposed in [18] to estimate the step length in walking and jogging situations.

The main contributions of this paper are summarized as follows:

- A novel filtering technique is proposed to eliminate on-ankle path loss outliers and keep the remaining as a reliable range with a pair of upper and lower thresholds. This range of path loss values was used to estimate the human step length in daily activities, such as walking and jogging;
- The distribution of the on-ankle path loss was revealed to follow a two-term Gaussian distribution, and the two thresholds lied on each side of its second hump;
- The thresholds can be determined mathematically. The upper threshold relates to the fitting equation of the second hump of the two-term Gaussian distribution, which was found as μ+0.5σ for an outdoor and μ+σ for an indoor environment. The lower threshold relates to the survival rate, which is located at the point where the survival rate of the measured data is 0.68;
- The proposed filtering technique resulted in an accurate estimation of the step length, with errors of only 10.15 mm and 4.40 mm for walking and jogging in an indoor environment, respectively, and only 4.81 mm and 10.84 mm for the same activities in an outdoor environment.

The rest of the paper is organized as follows. Section 2 reviews the related works. Section 3 describes the proposed system model. In Section 4, the experimental procedures are detailed. Section 5 presents the experimental results and analyses of the step length estimation accuracy in the indoor walking, indoor jogging, outdoor walking, and outdoor jogging situations. Section 6 concludes the paper. Finally, Section 7 states the limitations and the future works.

## 2. Related Works

Accurate estimation of the human step length is a challenging task, especially in human daily activities, due to the randomness of these activities. As a result, there are few research papers that explicitly address the problem of step length estimation, although the overarching topic of distance measurements has been intensively researched for general communication systems. These research papers are briefly reviewed as follows.

The researchers in [6] used cameras as additional sensors in pedestrian dead reckoning (PDR) to analyze step length and step frequency. Currently, PDR is a popular indoor localization method [19,20] due to the wide availability of smart devices. Cameras were also employed in [7] to track the motions of the person under test. The stride length can be estimated by detecting and extracting several pieces of perspective information related to predefined markers and edges. The experiment results implied that the camera-based method was a promising way to detect all steps when the user was moving slowly, especially in an indoor environment. Recently, the researchers in [21] proposed a machine-learning-based step length estimation algorithm with the use of cameras and smartphones. This research considered a systematic feature selection algorithm to determine the choice of user-specific parameters from a large collection. The mean absolute errors of the step length estimations were 3.48 cm and 4.19 cm for a known test person and an unknown test person, respectively. However, the above camera-based techniques are flexibility-constrained because the camera must be arranged at a certain place and has a limited horizon. Moreover, its accuracy may be reduced in fast-moving situations or by obstacles appearing between the cameras and the person under test.

The gait patterns can also be detected by infrared thermography, such as in [8], where the best accuracy was found to be 91%. However, the drawback is that lasers are not common in daily usage because of the training requirements, costly equipment, and the potential health concern for long-term exposure.

An inertial sensor can be utilized in an inertial measurement unit (IMU) to collect gait-related parameters, which then help to estimate the human step length. An IMU generally consists of an accelerometer, a compass, and a gyroscope. Currently, most smart devices have built-in inertial sensors. The smart device can be held in hand [22] or attached to the body, such as the pelvis [23], which provides useful information and helps position the point of interest. References [19,20] estimated the human stride length based on the data collected from inertial sensor measurements from a smartphone. The experimental results demonstrated that the step length can be estimated with an error rate of 4.63% for indoor scenarios. Considering a general step length of 0.7 m, the corresponding absolute error would be 3.24 cm. The error of step length estimation was reduced to 2% in [24] based on a back-propagation artificial neural network using an IMU that was placed on the foot. The research in [25] compared the accuracy of estimation between different placements of the IMUs. Firstly, this paper utilized only one inertial sensor on each shank, called the integrator-based method, providing an average accuracy of 91.21%. The accuracy was improved to 95.37% if two sensors were employed on each leg, namely the angle-based method. As a result, the maximum error was 11.26 cm and 5.51 cm for the integration and angle mode, respectively. Although the integrator-based method was simpler, the angle-based method achieved better accuracy in terms of step length estimation since it was not sensitive to the initial conditions and errors caused by double integration. However, experiments and analyses in the outdoors are still missing. Moreover, a major disadvantage of using IMUs is that they typically suffer from an accumulated error, which means the accuracy will be degraded over time.

Deep learning has been adopted to estimate human step length because it can learn the features of the data automatically and has shown excellent performance in different application domains with the cost of powerful computing facilities. The proposed deep-learning-based algorithm in [26] can adapt to different phone carrying ways and does not require individual stature information and spatial constraints. The average error of this method was 3.01%, which means if the actual step length was 0.7 m, then the corresponding error range was within 2.1 cm. Paper [27] defined a deep-learning-based framework with an activity recognition model to regress the user change in distance and step length. The average error of the proposed method was 2.1%, which was about 1.47 cm if the step length was 0.7 m. It is worth noting that the positions (e.g., handheld position or pocket position) of the smartphone also had a huge influence on the estimation by around 5% [28]. The researchers in [29] investigated human step length and step width using wearable sensors in a computer-assisted rehabilitation environment. The results showed that in a specific experimental environment, gait patterns could be detected and the mean absolute errors were 0.2396 cm and 1.92 cm, respectively. However, the data in this paper were collected using specific equipment under a specific environment, rather than normal indoor and outdoor propagation environments in daily human activities.

Therefore, in this paper, we aimed to propose a step length estimation technique that has high accuracy and is less-privacy-invasive, less-health-concerning, and less-space-confined than the aforementioned techniques, without requiring powerful computing facilities as the deep-learning-based ones.

Our previous work presented in [18] proposed an empirical path loss model to estimate the human step length in both indoor and outdoor scenarios under a static context rather than in a dynamic one. Therefore, this paper aimed to estimate human step length in daily activities. In particular, a novel filtering technique is proposed in this paper, which was used along with the hardware transceivers and the empirical path loss model developed in our previous work [18] to estimate human step length correctly in both walking and jogging activities in both indoor and outdoor environments.

## 3. System Model

In this paper, we adopted the transceivers and the experimental path loss model between two human ankles developed in [18]. The experimental path loss $PL_{OA}$ between two transceivers attached to the ankles of the person under test can be described as a modified free-space path loss model with a correction factor ΔPL (cf. (1) in [18]):

(1) $$PL_{OA}\left(\mathrm{dB}\right)=PL_{FS}+\Delta PL,$$

where $PL_{FS}$ (dB) is the free-space path loss and ΔPL (dB) is the correction factor, which accounts for the hardware non-linearity, multipath propagation, insertion, and mismatch losses. For the transceivers considered, the correction factor was empirically found as 10 dB [18]. Therefore, (1) can be written as:

(2) $$PL_{OA}\left(\mathrm{dB}\right)=PL_{FS}+10.$$

It is noted that the free-space path loss [30] is defined as:

(3) $$PL_{FS}\left(\mathrm{dB}\right)=20\log_{10}\left(\frac{4\pi d}{\lambda }\right),$$

where *d* (m) is the distance between the two antennas and λ (m) is the signal wavelength. From (2) and (3), this distance could be estimated as:

(4) $$d=\frac{\lambda }{4\pi }10^{\left(\frac{PL_{OA}\left(\mathrm{dB}\right)-10}{20}\right)}.$$

In the later analysis, we used this equation to calculate the human step length.

## 4. Experiment Setups

**Figure 1:**
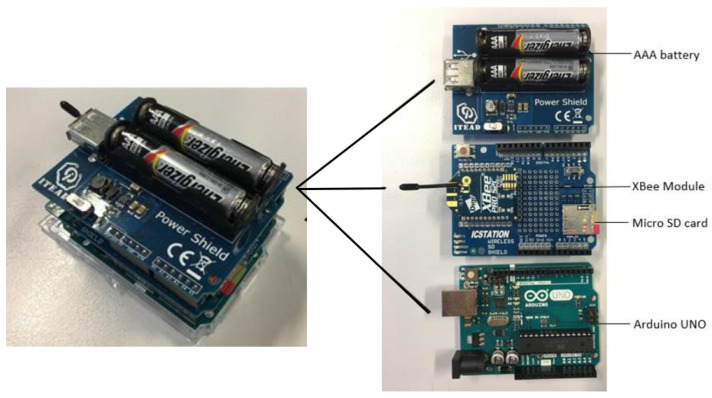
Components of the transceivers.

In this section, we detail our experimental settings. Similar to our previous work in [18], the Arduino Integrated Development Environment (IDE), XBee Configuration & Test Unit (X-CTU), Arduino UNO microprocessors, and XBee-PRO S2C wireless transceivers were employed in this experiment. The core communication technology used in the XBee-PRO S2C modules is the spreading spectrum technique regulated by the IEEE 802.15.4 standard for low-rate wireless personal area networks (LR-WPANs) [31]. In particular, each group of four data bits is mapped into one of 16 nearly orthogonal spreading sequences, each of which is 32 chips long. The resulting chip sequence is modulated on the radio-frequency carrier in the 2.4 GHz band by the offset quadrature phase shift keying (O-QPSK) modulation scheme. The components of the transceivers are depicted in Figure 1. The parameters were configured as follows: transmission power $P_{0}=0$ dBm and data rate 9600 bps. This is the most proper configuration of the developed transceivers for measuring the distance between two ankles, as discovered from our previous experiments in [18].

**Figure 2:**
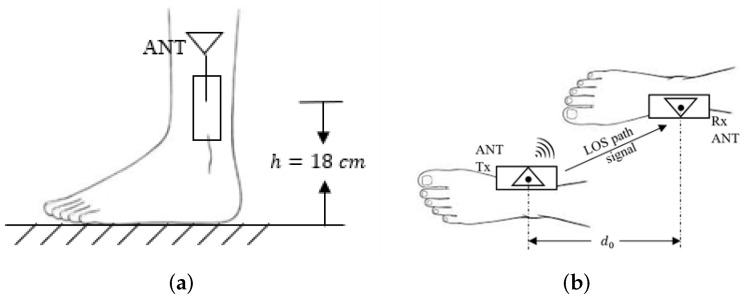
Schematic diagrams of the on-ankle transceivers. (**a**) Side view; (**b**) top view.

The transceivers were attached to the inner side of human ankles at the same height *h*, as shown in Figure 2. The distance between two antennas was regarded as the real step length $d_{0}$ (m). In our experiments, the transmitter and the receiver were placed on the medial side of the ankles of the subject under test in a way that the antennas faced each other, as shown in Figure 2b. This means that there existed an LOS path between the transceivers, even when the person under test was walking or jogging, and that there was no human body part appearing between them. As a result, this placement of equipment can eliminate the shadowing effect caused by any body parts. This intuitive prediction was confirmed in our previous work [18], where experiments were performed both off-body and on ankles to compare the shadowing effect. The results in [18] showed that the shadowing effect caused by the human body was negligible in our experiments.

The main purpose of this system was to transmit and receive continuous data packets to/from each other, and the assembled micro SD card in the receiver recorded the RSSI values continuously. From the RSSI values, the on-ankle path loss can be calculated as (cf. (5) in [18]):

(5) $$PL_{OA}\left(\mathrm{dB}\right)=P_{t}+RSSI,$$

where $P_{t}$ (dB) is the transmitted power. From (4) and (5), the distance between the two transceivers is:

(6) $$d=\frac{\lambda }{4\pi }10^{\left(\frac{P_{t}\left(\mathrm{dB}\right)+RSSI\left(\mathrm{dB}\right)-10}{20}\right)}.$$

**Figure 3:**
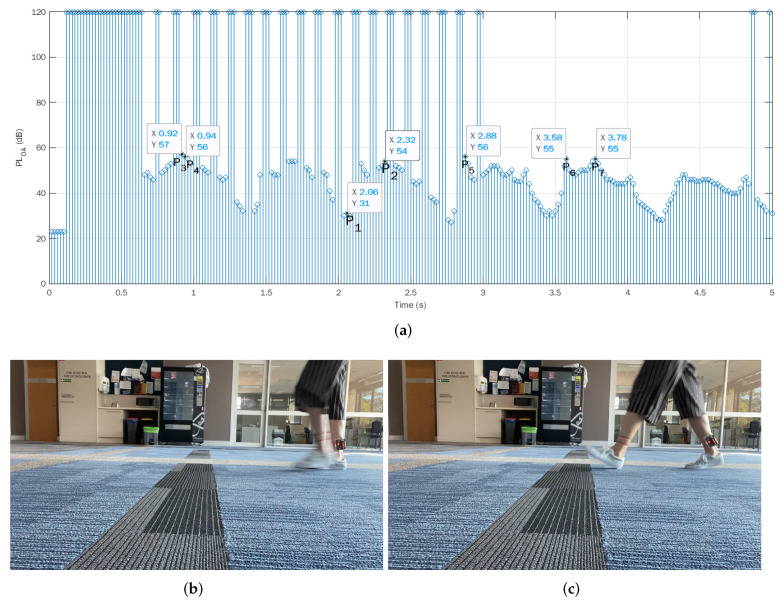
Trial indoor walking experiment. (**a**) On-ankle path loss at different time; (**b**) feet positions at $P_{1}$ (t = 2.08 s); (**c**) feet positions at $P_{2}$ (t = 2.32 s).

Following is a trial experiment of the indoor walking situation to explore the relationship between the measured path loss values and the positions of the two ankles. Figure 3a plots the on-ankle path loss over time. During the first 0.64 s, the transmitter and the receiver initialize themselves and synchronize with each other. Once the transmitter and the receiver are synchronized, it takes around 0.02 s for the hardware to measure and record each RSSI value into the micro-SD card, as shown in Figure 3b.

After the initial synchronization phase, the Arduino may encounter erroneous transmissions from time to time due to temporarily being out-of-synchronization. To cope with this, in our experiments, the Arduino UNO hardware was programmed in a way that, if an erroneous transmission occurs (i.e., the receiver does not receive the packet successfully), a very big value of path loss (120 dB was chosen in our experiments) would be recorded to the data file in the micro-SD card to flag this erroneous transmission. Thereby, in the later analysis, any erroneous transmission would be easily detected and omitted. As shown in Figure 3a, the temporary out-of-synchronization status was normally very short, and the Arduino UNOs could quickly synchronize again with each other. Hence, in general, the Arduino UNO transceivers were relatively stable and reliable.

Because of the modest computation capability of the Arduino UNO, the transceivers in our experiments were programmed to only transmit and receive data packets to record the RSSI values in order to avoid any unnecessary delay. Processing of the raw data was performed offline on a computer instead. It is also noted that we aimed to estimate the average step length over a certain period, rather than outputting the instant estimated step length values, to mitigate the randomness in the measurement process. As a result, the processing time of our algorithm had a negligible effect on the RSSI calculations.

It was observed that the measured path losses had a periodical pattern. To explore the meaning of the peaks and troughs of the path loss, let us consider two points $P_{1}$(2.08 s, 30 dB) and $P_{2}$(2.32 s, 54 dB) from the plot, where $P_{1}$ is at a trough and $P_{2}$ is the following peak. A video of the footage was captured in tandem with the path loss measurements. Based on the time stamps, we obtained the corresponding video frames, which corresponded to $P_{1}$ and $P_{2}$, as shown in Figure 3b,c. In Figure 3b, two feet are aligned with each other. In other words, at $P_{1}$, the distance between two ankles is the shortest, which indicates the pedestrian has moved the left leg from behind to the middle position and is about to step forward. Hence, a step is half-finished at the bottom points of Figure 3a. The step is fully finished in Figure 3c. The ankles are at the largest distance from each other, where $P_{2}$ is located. This means the peak path loss value at $P_{2}$ in the time duration [2.08 s, 2.32 s] coincidentally corresponds to the step length. Note that $PL_{OA}=54$ dB is not the global largest value of path loss in Figure 3a. For example, the peak path loss values at the points $P_{3}$–$P_{7}$ at the time instants 0.92 s, 0.94 s, 2.88 s, 3.58 s, and 3.78 s were even bigger than 54 dB. In other words, the path loss corresponding to the step length is expected to be in a high range of the path loss values, but not necessarily the largest value. Hence, to find the step length, it was necessary to examine the histogram of the experimental data.

**Figure 4:**
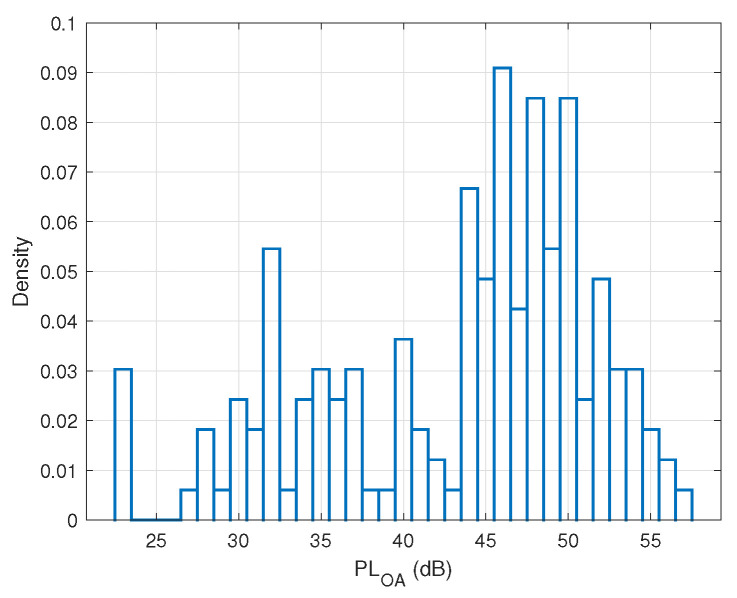
The probability histogram of the trial indoor walking experiment.

**Figure 5:**
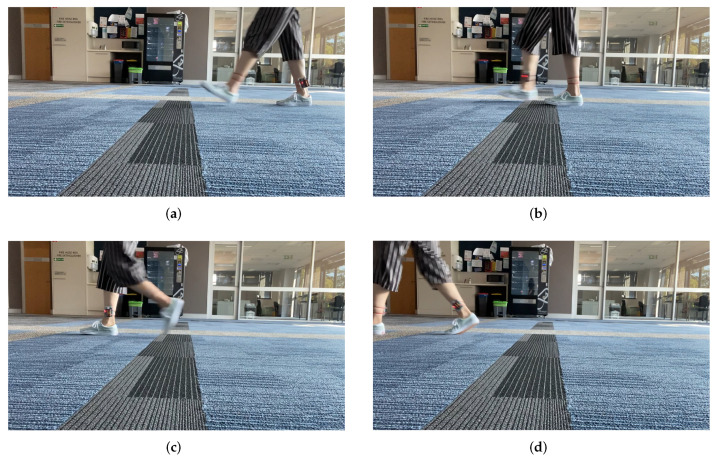
Feet positions at different time stamps of the indoor walking experiment. (**a**) t = 2.22 s ($PL_{OA}=46$ dB); (**b**) t = 2.80 s ($PL_{OA}=46$ dB); (**c**) t = 3.12 s ($PL_{OA}=50$ dB); (**d**) t = 3.72 s ($PL_{OA}=50$ dB).

The bar chart in Figure 4 depicts the probability histogram of the on-ankle path loss in this trial experiment. Clearly, the histogram shows a two-hump shape with the most likely path loss occurring at the peak density $PL_{OA}\approx 46$ dB. The first, smaller hump corresponds to the half-finished steps, i.e., when the two feet are about to cross each other. The second, bigger hump corresponds to the events when the two feet are likely most separated from each other. The step length (i.e., the maximum distance between the two transceivers) may occur somewhere around the peak density rather than always at the peak density in the histogram. To demonstrate this point, let us consider the two different moments t=2.22 s and t=2.80 s when the path loss of 46 dB took place (cf. Figure 5a,b). These two figures suggest that, although the on-ankle path losses at these time instants were the same and both corresponded to the peak density in the histogram, the feet of the person under test were not in the identical posture. This means that the path loss corresponding to the peak density did not always correspond to the step length due to the randomness of the propagation channel. This observation is confirmed again in Figure 5c,d, where we show the two maximum distance events at the time instants t=3.12 s and t=3.72 s when $PL_{OA}\approx 50$ dB. The path loss $PL_{OA}\approx 50$ dB corresponds to the second maximum density, rather than the peak one in Figure 4.

From the aforementioned observations, we conjectured that the human step length can be estimated within a certain range around the peak density of the histogram. This is because the actual step length may occur before or after the peak density due to the randomness of the propagation channel caused by the dynamic motions of the person under test. Therefore, in the following experiments, we propose a filtering technique to discard outlier data to form a range of reliable path loss values for estimating the step length. The accuracy analyses are also mentioned in the next section.

## 5. Experimental Results and Analysis

**Figure 6:**
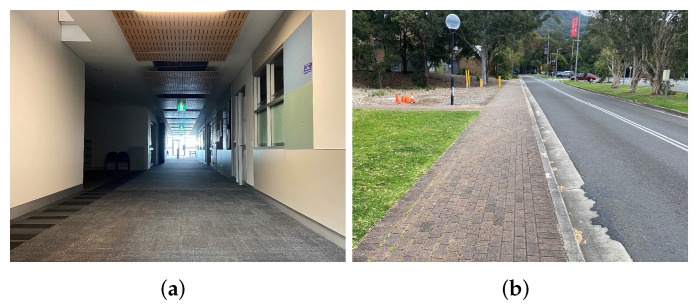
Experimental environments. (**a**) Indoors; (**b**) outdoors.

In this section, experiments were conducted in four dynamic scenarios, including indoor walking, outdoor walking, indoor jogging, and outdoor jogging. The indoor experiments were carried out in a corridor of a building, while the outdoor ones were conducted along some pavement, which can be seen as an open area in Figure 6. The participant walked or jogged along a straight path with a length of 35.7 m. There were 50 steps and 38 steps in the walking and jogging scenarios, respectively. Therefore, the real average step length for walking was $d_{0w}=35.7÷50=0.7140$ m, while for jogging, it was $d_{0j}=35.7÷38=0.9395$ m. In each scenario, the experiments were carried out 10 times with over 1500 data in each dataset. Altogether, there were more than 15,000 data for each scenario. In our previous work [18], we derived the empirical path loss model for the wireless channel between the two ankles in a static situation, as shown in (1). As mentioned above, there existed randomness of the path loss in dynamic situations where the person under test was walking or jogging. Thus, we propose a filtering technique to apply along with the empirical model in (1) in order to eliminate the on-ankle path loss outliers. The resulting ranges of on-ankle path loss were then used to estimate the step length in the four motion scenarios. The following subsections are the experiment results and analyses for the four motion circumstances.

### 5.1. Empirical Threshold Pair

**Figure 7:**
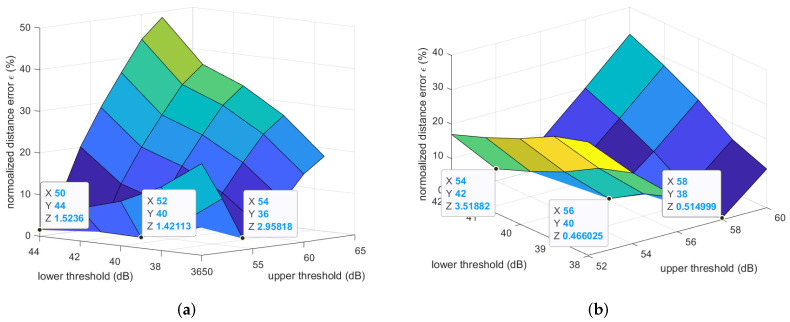
Normalized step length estimation errors of the indoor experiments. (**a**) Indoor walking; (**b**) indoor jogging.

We propose a novel filtering technique to filter out the path loss outliers by setting a threshold pair, which consisted of an upper threshold and a lower threshold. As these two thresholds work together, we found both thresholds simultaneously. As shown in Figure 3 and Figure 5, the path loss for the step length could be neither the maximum path loss value nor the path loss value corresponding to the peak density of the histogram. This was because the randomness of the propagation channel was caused by the dynamic movements of the person under test. Thus, it is important to consider a suitable range of the path loss values that might possibly correspond to the maximum distance between two ankles. To this end, based on the collected datasets, we first examined different combinations of the lower bound and the upper bound of this range to find the pair of boundaries that minimized the error between the average estimated step length and the true step length. The path loss values higher than the upper threshold or lower than the lower threshold were considered as outlier values. Figure 7 demonstrates the normalized errors of the step length estimations in the indoor walking and indoor jogging scenarios for different lower and upper thresholds. The relative (or normalized) error ϵ in percentage is defined as:

(7) $$\epsilon =\frac{|\bar{d}-d_{0i}|}{d_{0i}}\times 100\%,$$

where $\bar{d}$ is the average estimated distance between two ankles under a certain experimental scenario, which involves 10 datasets, $d_{0i}$ is the real step length, and *i* is either *w* for the walking scenario or *j* for the jogging scenario. Figure 7 shows that the (lower, upper) threshold pairs of (40 dB, 52 dB) and (40 dB, 56 dB) resulted in the average estimated step lengths being the closest to the true step lengths (i.e., the smallest normalized error ϵ) in the indoor walking and indoor jogging scenarios, respectively. Along with Figure 7, Table 1 and Table 2 show in more detail the estimated step length (cf. (4)), averaged over all ten datasets for some different pairs of the (lower, upper) thresholds for the indoor walking and indoor jogging scenarios. In each cell of the table, there are three numbers. The average estimated step length in meters, which is the average result based on 10 experimental datasets, is located outside of the brackets. Following in the brackets are the average absolute error in millimeters and the average relative error in percentage, respectively.

The average absolute error was calculated as $|\bar{d}-d_{0i}|$. Table 1 and Table 2 confirm further the observation gained from Figure 7 that the best pairs of (lower, upper) thresholds of the path losses were (40 dB, 52 dB) and (40 dB, 56 dB) for the indoor walking and indoor jogging cases, respectively. The average absolute and normalized estimation errors were just 10.15 mm and 1.42% for the indoor walking case, while these numbers were 4.40 mm and 0.47% for the indoor jogging case.

**Figure 8:**
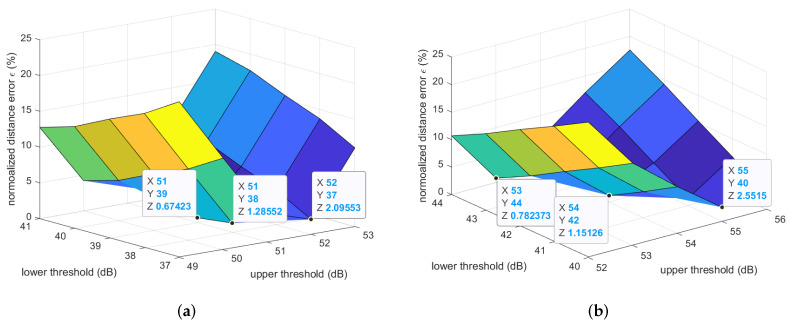
Normalized step length estimation errors of the outdoor experiments. (**a**) Outdoor walking; (**b**) Outdoor jogging.

Similarly, Figure 8 and Table 3 and Table 4 clearly show that the best (lower, upper) thresholds of the path losses used for estimating the average step length in the outdoor walking and jogging scenarios were (39 dB, 51 dB) and (42 dB, 54 dB), respectively. The average absolute and relative estimation errors for the former case were just 4.81 mm and 0.67%, while they were 10.84 mm and 1.15% for the latter one.

**Figure 9:**
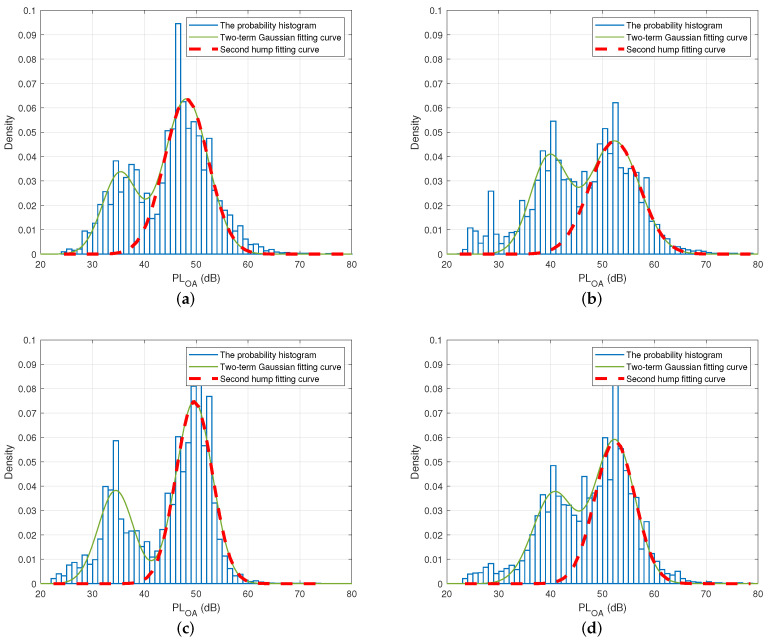
The probability histogram, the two-term Gaussian distribution, and the fitting curve of the second hump for the indoor and outdoor experiments. (**a**) Indoor walking; (**b**) indoor jogging; (**c**) outdoor walking; (**d**) outdoor jogging.

It is noted that the estimation error in the indoor walking scenario was higher than that in the indoor jogging one. This can be explained as follows. In general, one might expect that the error of the walking scenarios is smaller than that of the jogging ones as walking is a slower and more stable activity than jogging. This expectation was confirmed from the experimental results of the outdoor scenarios, where the errors for outdoor walking and jogging were 4.81 mm and 10.84 mm, respectively. However, this expectation may not always be the case for an indoor environment since there are more multipaths indoors than outdoors. Because walking takes a longer time than jogging to complete a step, when multipath propagation occurred, more affected RSSI (thus path loss) values during that step were recorded to the dataset in the walking scenario than in the jogging one. As a result, the histogram of the path loss dataset collected for the indoor walking scenario may have some (local) peaks that were far more distinct from the remaining non-peak values, compared to the indoor jogging case. This phenomenon can be observed in Figure 9a (mentioned later in Section 5.2), where the density of the path loss value of 46 dB was much more prominent than other non-peak values, while the local peaks in Figure 9b are less prominent compared to their surrounding values. This led to a slightly worse accuracy in average step length estimation in the indoor walking compared to the indoor jogging.

### 5.2. Upper Threshold Analysis

The data analyses mentioned in Section 5.1 are critical as they allowed us to devise the novel filtering technique, which is detailed below.

In order to formulate the thresholds mathematically, we firstly depict the probability histogram for all the datasets (around 15,000 data) collected in each experimental scenario, as shown in Figure 9. The probability histogram of the measured on-ankle path loss is represented by blue bars. It is noted that the plotted histogram has two humps, which correspond to the half-finished step, where the two feet are about to pass each other, and the fully finished step, when the two feet are most apart from each other, respectively. The plotted histogram can be well approximated by the probability density function (PDF) of a two-term Gaussian distribution model via the curve-fitting process indicated by the solid green curve in Figure 9 with the general PDF equation:

(8) $$\begin{array}{cc} f(x) & =f_{1}\left(x\right)+f_{2}\left(x\right)\\ \; & =a_{1}e^{-{\left(\frac{x-b_{1}}{c_{1}}\right)}^{2}}+a_{2}e^{-{\left(\frac{x-b_{2}}{c_{2}}\right)}^{2}}, \end{array}$$

where $f_{k}\left(x\right)=a_{k}e^{-{\left(\frac{x-b_{k}}{c_{k}}\right)}^{2}}$, $a_{k}$ is the amplitude, $b_{k}$ is the centroid, and $c_{k}$ relates to the peak width of this Gaussian distribution (k=1, 2). These coefficients can be found from the curve fitting of the two-term Gaussian distribution model. Ideally, the step length is related to the maximum on-ankle path loss. However, due to the randomness of the propagation channel, the actual step length may correspond to a non-peak path loss around the peak of the second hump. This means that the pair of the (lower, upper) thresholds should capture a suitable range of the path loss values around the peak of the second hump. The values bigger than the upper threshold or smaller than the lower threshold were considered as outliers for estimating the path loss that corresponds to the step length. To capture the suitable window of the possible path loss values for estimating the step length, intuitively, the upper threshold should be located somewhere at the right slope of the second hump, while the lower threshold lies somewhere at the left slope of the second hump, i.e., in between the first hump and the second hump.

From Figure 9, it is observed that the impact of the first hump on the right slope of the second hump was negligible. Thus, we can extract the second hump and approximate its right slope by the Gaussian distribution:

(9) $$f_{2}\left(x\right)=a_{2}e^{-{\left(\frac{x-b_{2}}{c_{2}}\right)}^{2}}.$$

This observation is confirmed in Figure 9a, where the bell-shaped red dashed curve representing the Gaussian distribution in (9) coincides with the right slope of the second hump of the two-term Gaussian distribution. As a result, we can obtain the mean μ and the standard deviation σ of the second hump based on the above Gaussian distribution in (9) as:

(10) $$\mu =b_{2},$$

(11) $$\sigma =\frac{c_{2}}{\sqrt{2}}.$$

The above observations and analyses hold for all indoor/outdoor walking and indoor/outdoor jogging cases, as shown in Figure 9a–d.

The curve fitting parameters $a_{2}$, $b_{2}$, $c_{2}$, μ, and σ for the second hump in the four scenarios can be found in Table 5. Since the path loss, which corresponds to the step length, is a random variable, its upper threshold should be determined as a function of both the mean value μ and the standard deviation value σ of the second term of the two-term Gaussian distribution in (9). This philosophy is similar to the well-known concept of calculating the retransmission timeout (RTO) on the Internet where the RTO is the function of both the mean value of the round-trip time (RTT) and its deviation value.

Table 6 presents the values of function μ+kσ(k=0,0.5,1,1.5,2),and the corresponding difference, denoted as Δ (dB), between these values and the upper thresholds, which were worked out empirically from the actual measured data in Section 5.1. Table 6 clearly shows that the empirical upper thresholds in the indoor walking and jogging scenarios were both very well approximated by μ+σ with the differences Δ of only 0.3319 dB and 1.1391 dB, respectively. This finding makes sense because the upper threshold is equal to the mean path loss value μ plus a margin, which is equal to the standard deviation σ in this case.

Similarly, the empirical upper thresholds in the outdoor walking and jogging scenarios were both very close to μ+0.5σ with the difference Δ of merely 0.3541 dB and 0.3208 dB, respectively. The upper thresholds in the two indoor cases were higher than those in the outdoor scenarios due to the fact that there were more multipaths indoors than outdoors; thus, the actual path loss that corresponds to the step lengths might vary more widely around its mean value.

### 5.3. Lower Threshold Analysis

**Figure 10:**
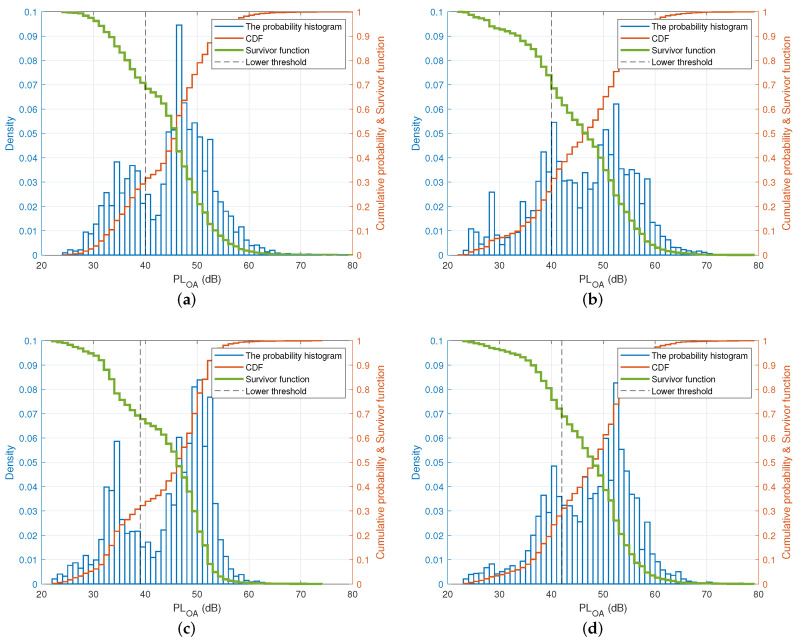
The probability histogram, the CDF, and the survivor function with respect to the lower threshold for the indoor and outdoor experiments. (**a**) Indoor walking (lower threshold = 40 dB); (**b**) indoor jogging (lower threshold = 40 dB); (**c**) outdoor walking (lower threshold = 39 dB); (**d**) outdoor jogging (lower threshold = 42 dB).

As mentioned in Section 5.2, the lower threshold was located between the first hump and the second hump of the two-term Gaussian distribution, which means its value would be affected by both humps. Therefore, it was impossible to analyze the lower threshold based on a single hump as for the upper bound mentioned above. Thus, other techniques should be used to analyze the lower threshold. One of the possible techniques is based on the cumulative distribution function (CDF) or the survival function. The survival function is complementary to the CDF. It indicates the probability of the path loss value greater than or equal to a certain value. Figure 10 depicts the probability histogram, the CDF (the red curves), and the survival function (the bold green curves) of the measured path loss data for all four scenarios together with the lower thresholds (the black dashed lines), which were empirically found to be 39 dB and 42 dB in the outdoor walking and jogging cases, respectively, and 40 dB in the indoor cases, as detailed in Section 5.1.

Figure 10 reveals an interesting fact that the intersections between the empirical lower thresholds and the survival curves were around 0.68 in all four cases. In other words, the measured path loss value was bigger than or at least equal to the value of the lower threshold 68% of the time in all four scenarios. Path loss values between the lower threshold and the upper one should be considered as the potential path losses corresponding to the step lengths. Based on the above empirical measurements and statistical analyses, we deduced that the lower threshold can be numerically found as the corresponding path loss value when the survival rate reaches 0.68.

## 6. Conclusions

This paper estimated the human step length in daily activities based on our developed wearable transceivers and the RSSI method. We conducted experiments for both walking and jogging activities in both indoor and outdoor environments. By analyzing the statistical properties of the collected datasets, for the first time, we proposed a filtering method to set up the lower and upper thresholds in order to eliminate the path loss outliers. The resulting range of path loss values between the two thresholds was used to estimate the step length. Mathematically, the upper threshold for an indoor environment was μ+σ, while this value for an outdoor scenario was μ+0.5σ. The lower threshold relates to the survival function of the experimental datasets. This threshold was found numerically to be the path loss value where the survival rate was around 0.68 for both the indoor and outdoor environments and for both the walking and jogging activities. Our experiments showed that the step length can be accurately estimated with errors of only 10.15 mm and 4.40 mm for the indoor walking and jogging activities and errors of 4.81 mm and 10.84 mm for the outdoor walking and jogging activities, respectively.

## 7. Limitations and Future Works

The experimental results showed that the proposed system along with the proposed technique can estimate the average human step length with a sub-centimeter error. However, a limitation of this project is that we need to collect the dataset for the whole intended period of time, then proceed to the offline data processing phase, rather than processing data to estimate the step length and updating this estimation in a continuous manner while the person under test is moving. Overcoming this limitation is the motivation for our future work. More specifically, to guarantee both accuracy and efficiency, instead of waiting for the whole dataset to be collected, we may apply the weighted moving average algorithm to continuously estimate the average step length and keep updating this estimation over a shorter period of time. In this way, the dynamic essence of human activities will be captured more accurately than the simple averaging technique. In addition, we may consider a hybrid RSSI-based technology [32], such as adopting an IMU in the existing RSSI-measuring system along with an RSSI/IMU data fusion approach, to further improve the precision and robustness of the step length estimation.

## Figures and Tables

**Table 1:** Estimation of the step length (m) in the indoor walking scenario for different pairs of upper threshold $PL_{u}$ (dB) and lower threshold $PL_{l}$ (dB). Two values in the brackets of each table cell are the corresponding absolute estimation error (mm) and relative estimation error (%), respectively.

	${\mathrm{PL}}_{l}$ (dB)	50	52	54	56	58
${\mathrm{PL}}_{u}$ (dB)						
**36**		0.5556 (158.39, 22.18)	0.6206 (93.41, 13.08)	0.6929 (21.12, 2.96)	0.7452 (31.21, 4.40)	0.7896 (75.55, 10.58)
**38**		0.6040 (109.96, 15.40)	0.6701 (43.86, 6.14)	0.7448 (30.83, 4.32)	0.7997 (85.65, 12.00)	0.8466 (132.56, 18.57)
**40**		0.6372 (76.83, 10.76)	**0.7039** **(10.15, 1.42)**	0.7802 (66.19, 9.27)	0.8368 (122.82, 17.20)	0.8856 (171.60, 24.03)
**42**		0.6577 (56.30, 7.88)	0.7251 (11.14, 1.56)	0.8029 (88.92, 12.45)	0.8610 (146.98, 20.59)	0.9112 (197.21, 27.62)
**44**		0.7031 (10.88, 1.52)	0.7746 (60.60, 8.49)	0.8578 (143.82, 20.14)	0.9207 (206.66, 28.94)	0.9755 (261.53, 36.63)

**Table 2:** Estimation of the step length (m), absolute estimation error (mm), and relative error (%) in the indoor jogging scenario for different pairs of upper threshold $PL_{u}$ (dB) and lower threshold $PL_{l}$ (dB).

	${\mathrm{PL}}_{l}$ (dB)	52	54	56	58	60
${\mathrm{PL}}_{u}$ (dB)						
**38**		0.6282 (311.28, 33.13)	0.7458 (193.70, 20.62)	0.8461 (93.37, 9.94)	0.9443 (4.81, 0.51)	1.0450 (105.55, 11.23)
**39**		0.6557 (283.77, 30.20)	0.7758 (163.66, 17.42)	0.8785 (61.03, 6.50)	0.9791 (39.59, 4.21)	1.0825 (143.03, 15.22)
**40**		0.7050 (234.47, 24.96)	0.8288 (110.70, 11.78)	**0.9351** **(4.40, 0.47)**	1.0397 (100.21, 10.67)	1.1477 (208.21, 22.16)
**41**		0.7450 (194.48, 20.70)	0.8708 (68.73, 7.32)	0.9795 (40.01, 4.26)	1.0870 (147.48, 15.70)	1.1984 (258.86, 27.55)
**42**		0.7795 (160.00, 17.03)	0.9064 (33.09, 3.52)	1.0170 (77.51, 8.25)	1.1268 (187.30, 19.94)	1.2410 (301.49, 32.09)

**Table 3:** Estimation of the step length (m), absolute estimation error (mm), and relative error (%) in the outdoor walking scenario for different pairs of upper threshold $PL_{u}$ (dB) and lower threshold $PL_{l}$ (dB).

	${\mathrm{PL}}_{l}$ (dB)	49	50	51	52	53
${\mathrm{PL}}_{u}$ (dB)						
**37**		0.5584 (155.56, 21.79)	0.6225 (91.54, 12.82)	0.6851 (28.93, 4.05)	0.7290 (14.96, 2.10)	0.7925 (78.54, 11.00)
**38**		0.5798 (134.16, 18.79)	0.6430 (71.02, 9.95)	0.7048 (9.18, 1.29)	0.7484 (34.40, 4.82)	0.8118 (97.79, 13.70)
**39**		0.5952 (118.81, 16.64)	0.6576 (56.41, 7.90)	**0.7188** **(4.81, 0.67)**	0.7622 (48.15, 6.74)	0.8254 (111.38, 15.60)
**40**		0.6125 (101.55, 14.22)	0.6740 (40.05, 5.61)	0.7345 (20.46, 2.87)	0.7775 (63.52, 8.90)	0.8406 (126.59, 17.73)
**41**		0.6229 (91.06, 12.75)	0.6839 (30.07, 4.21)	0.7440 (30.02, 4.21)	0.7869 (72.94, 10.22)	0.8499 (135.95, 19.04)

**Table 4:** Estimation of the step length (m), absolute estimation error (mm), and relative error (%) in the outdoor jogging scenario for different pairs of upper threshold $PL_{u}$ (dB) and lower threshold $PL_{l}$ (dB).

	${\mathrm{PL}}_{l}$ (dB)	52	53	54	55	56
${\mathrm{PL}}_{u}$ (dB)						
**40**		0.7082 (231.29, 24.62)	0.7990 (140.54, 14.96)	0.8599 (79.64, 8.48)	0.9155 (24.00, 2.55)	0.9651 (25.58, 2.72)
**41**		0.7420 (197.45, 21.02)	0.8338 (105.70, 11.25)	0.8952 (44.30, 4.72)	0.9515 (11.98, 1.28)	1.0018 (62.32, 6.63)
**42**		0.7745 (164.97, 17.56)	0.8669 (72.56, 7.72)	**0.9287** **(10.84, 1.15)**	0.9855 (45.95, 4.89)	1.0365 (96.96, 10.32)
**43**		0.8085 (130.98, 13.94)	0.9013 (38.16, 4.06)	0.9632 (23.74, 2.53)	1.0205 (80.98, 8.62)	1.0721 (132.64, 14.12)
**44**		0.8391 (100.41, 10.69)	0.9321 (7.38, 0.79)	0.9941 (54.60, 5.81)	1.0517 (112.21, 11.94)	1.1040 (164.46, 17.51)

**Table 5:** Coefficients for the second hump-fitting equation.

	Indoor Walking	Indoor Jogging	Outdoor Walking	Outdoor Jogging
$a_{2}$	0.06369	0.04639	0.07489	0.05866
$b_{2}$	48.0900	52.3400	49.6100	52.3900
$c_{2}$	5.9990	6.7870	4.9330	5.4610
μ	48.0900	52.3400	49.6100	52.3900
σ	4.2419	4.7991	3.4882	3.8615

**Table 6:** Absolute difference Δ (dB) between the function μ+kσ and the empirical upper threshold (indoor walking: 52 dB; indoor jogging: 56 dB; outdoor walking: 51 dB; outdoor jogging: 54 dB).

	Indoor Walking	Indoor Jogging	Outdoor Walking	Outdoor Jogging
μ(Δ)	48.0900 (3.9100)	52.3400 (3.6600)	49.6100 (1.3900)	52.3900 (1.6100)
μ+0.5σ(Δ)	50.2110 (1.7890)	54.7396 (1.2604)	**51.3541 (0.3541)**	**54.3208 (0.3208)**
μ+σ(Δ)	**52.3319 (0.3319)**	**57.1391 (1.1391 )**	53.0982 (2.0982)	56.2515 (2.2515)
μ+1.5σ(Δ)	54.4529 (2.4529)	59.5387 (3.5387)	54.8422 (3.8422)	58.1823 (4.1823)
μ+2σ(Δ)	56.5738 (4.5738)	61.9383 (5.9383)	56.5863 (5.5863)	60.1130 (6.1130)

## Data Availability

The data presented in this study are available upon request from the corresponding author.

## Abbreviations

The following abbreviations are used in this manuscript:

CDF	Cumulative distribution function
IDE	Integrated Development Environment
IMU	Inertial measurement unit
LOS	Line-of-sight
LR-WPAN	Low-rate wireless personal area network
O-QPSK	Offset quadrature phase shift keying
PDR	Pedestrian dead reckoning
RF	Radio frequency
RSSI	Received signal strength indicator
X-CTU	XBee Configuration & Test Unit
